# Combined photodynamic-chemotherapy investigation of cancer cells using carbon quantum dot-based drug carrier system

**DOI:** 10.1080/10717544.2020.1765431

**Published:** 2020-05-18

**Authors:** Xin Li, Kandasamy Vinothini, Thiyagarajan Ramesh, Mariappan Rajan, Andy Ramu

**Affiliations:** a Department of Medical Oncology, Xinxiang Central Hospital, The Fourth Clinical College of Xinxiang Medical University, Xinxiang, Henan, China;; b Department of Inorganic Chemistry, School of Chemistry, Madurai Kamaraj University, Madurai, India;; c Department of Natural Products Chemistry, School of Chemistry, Biomaterials in Medicinal Chemistry Laboratory, Madurai Kamaraj University, Madurai, India;; d Department of Basic Medical Sciences, College of Medicine, Prince Sattam Bin Abdulaziz University, Al-Kharj, Kingdom of Saudi Arabia

**Keywords:** 5-Aminolevulinic acid, breast cancer, carbon quantum dots, combined therapy, drug delivery

## Abstract

The combined chemotherapy and photodynamic therapy have significant advantages for cancer treatments, which have higher therapeutic effects compared with other medicines. Herein, we focused on the synthesis of carbon quantum dot (CQD) based nanocarrier system. CQD and 5-aminolevulinic acid (5-ALA) were conjugated with mono-(5-BOC-protected-glutamine-6-deoxy) β-cyclodextrin (CQD-Glu-β-CD) moiety, and finally, the anticancer chemotherapy doxorubicin (DOX) drug was loaded in the 5-ALA-CQD-Glu-β-CD system. The stepwise physicochemical changes for the preparation of the DOX loaded 5-ALA-CQD-Glu-β-CD system were investigated by Fourier transform infrared (FT-IR) spectroscopy, X-ray diffraction (XRD), transmission electron microscopy (TEM), atomic force microscopy (AFM), and Raman fluorescence spectroscopy. The encapsulation efficiency of DOX in 5-ALA-CQD-Glu-β-CD was observed at ∼83.0%, and the loading capacity of DOX is ∼20.37%. The *in vitro* releasing of DOX and 5-ALA was observed through the UV–vis spectroscopy by the *λ*_max_ value of 487 nm and 253 nm, respectively. By the investigation against the breast MCF-7 cancer cells, the high cytotoxicity and morphological changes of cancer cells were observed by the treating of DOX/5-ALA-CQD-Glu-β-CD. The generation of reactive oxygen species (ROS) upon 635 nm (25 mW cm^−2^) for 15 min laser irradiation-induced improved the therapeutic effects. *In vitro* cellular uptake studies recommend the synthesized DOX/5-ALA-CQD-Glu-β-CD nanocarrier could significantly enhance the cell apoptosis and assist in the MCF-7 cell damages. The result suggests a multifunctional therapeutic system for chemo/photodynamic synergistic effects on cancer therapy.

## Graphical Abstract

**Figure:**
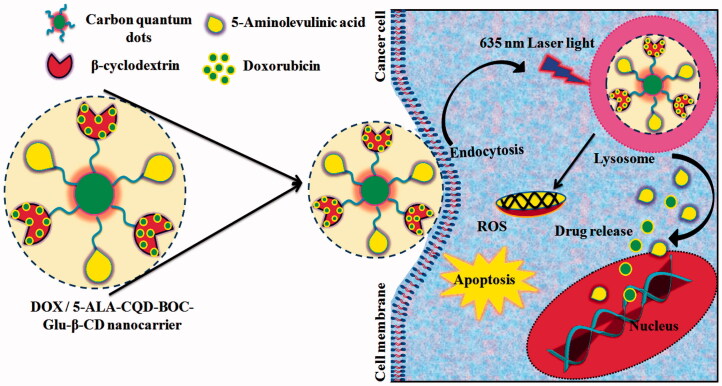

## 1. Introduction

Cancer is an extremely complex disease that affects human health, and it implicates multiple signaling pathways, making the therapy a highly challenging one (Gaio et al., 2019). Chemotherapy is one of the most frequently applied treatments of cancer. Many potent chemo drugs are used in clinical trials, and there are some drawbacks such as lack of selectivity, poor bioavailability, toxic effect against the healthy cells, etc. (Zhang et al., 2017). Notably, the resistance cancers cells have limited impact against the chemo-drugs that begin to play a critical role in cancer treatment (Wang et al., 2011). Recently, the combination of chemo and photodynamic therapy (PDT) improved the therapeutic effects in multidrug-resistant cancer cells. Under an applicable photo-irradiation, photosensitizer can be stimulated to the ground states to excited triplet states, at the same time which can consequently transfer energy to molecular oxygen, and which can lead to the formation of singlet oxygen (^1^O_2_) to kill the cancer cells. The 5-aminolevulinic acid (5-ALA) is an emerging compound and broadly utilized as a PS molecule in the treatment of different types of cancers (Mahmoudi et al., 2019). The mechanism of the 5-ALA molecules in contradiction of cancer cell inhibition is a protoporphyrin IX (PpIX) precursor for the heme synthesis pathway in a cell organ and mitochondria (Tewari & Eggleston, 2018). The polysaccharide-based DOX and 5-ALA loaded system mediate the photodynamic combined chemotherapy that induces more amount of apoptosis on breast cancer cells (Wang et al., 2019).

The photosensitizer combined chemo-drugs have more considerable attention in cancer treatments. The laser coupled photodynamic effect of Ce6 and DOX loaded micelles shows active cytotoxic valid on MDA-MB-231 cell line with single treatment alone (Zhang et al., 2017). For reaching the synergetic effect of chemo and photobiomolecule, various systems investigated with the assortment of polymers, carbon-based materials, and inorganic hydrate materials, etc. (Hongyu et al. 2020).

The carbon quantum dot (CQD) has fascinated widespread interest because of their excellent biocompatibility, low toxicity, good disperse ability in the water medium. It has unique photoluminescence properties since it is widely used for a nano-based drug delivery system for various diseases drug, bioactive compounds, and gene delivery (Zhu et al., 2013; Hallaj et al., 2015; Namdari et al., 2017; Chen et al., 2019; Xian Wen et al., 2017). The CQD has manifest some desirable physical and chemical properties like higher surface area, and better surface passivation properties for the conjugation of biologically enhancer molecules like targeting ligands, bioavailability enhancing moieties and linkers for carrier system (Wu et al., 2013; Li et al., 2015; Kang et al., 2018). Equally, β-cyclodextrin (β-CD) is an essential polysaccharide derivative. It consists of cyclic oligosaccharide of a seven-membered ring of the α-d-glucose unit connected via α-(1-4) linkages through an internal hydrophobic cavity and external hydrophilic cavity. It has been frequently used as host molecules to build a host–guest drug loading to the formation of host–guest inclusion complexes. The β-CD was ascribed to weak interaction like hydrophobic effect, van der Waals forces, and hydrogen bonding, hence the guest molecule was bound into the hydrophobic cavity of β-CD (Prabha & Raj, 2016; Caldera et al., 2017). N-doped carbon dots were used to drug delivery and bioimaging application. The N-doped carbon dots were conjugated with paclitaxel through labile ester bond conjugation, which improves the solubility of NCND-PTX. Further, NCND-PTX shows efficient cell uptake, cell apoptosis, and intracellular imaging of cancer cell lines compared to free PTX.

Since to keep in mind the importance of a combined drug carrier system, the β-CD functionalized CQD-based drug carrier system was developed. The anticancer chemo drug (DOX) and photosensitizer (5-ALA) drug were delivered to inhibit breast cancer cells via chemo-photodynamic treatment. In this work, the CQD acts as a dual role in the carrier system as nanoplatforms for drug carrier and fluorescent nature for the diagnosis of cancer cells. The system will give higher therapeutic and synergetic effects for breast cancer cells by the inhibition of DOX, and 5-ALA could generate the reactive oxygen species (ROS) to execute an irradiation impact on cancer cells. To best of our knowledge, this is a new study to suppress the cancer disease by utilizing the carbon-based drug carrier system to fulfill the needs of cancer treatment, and it will act as a potential drug carrier for the delivery of anticancer drugs.

## 2. Materials and methods

### 2.1. Materials

Citric acid, urea, glutamine, β-CD, *p*-toluene sulfonyl chloride, 1-ethyl-3-(3-dimethyl aminopropyl) carbodiimide hydrochloride (EDC⋅HCl) and *N-*hydroxy succinimide (NHS) were purchased from HIMEDIA Pvt. Ltd. (Mumbai, India). Doxorubicin (DOX), 5-ALA, di-tert-butyl dicarbonate (BOC), *N, N*-dimethyl formamide (DMF), and acetone were obtained from Sigma-Aldrich (Bangalore, India). All chemicals were of analytical grade and used directly. Double distilled water was used throughout experiments.

### 2.2. Synthesis of carbon quantum dot

The CQDs were synthesized according to a earlier reported procedure (Liu et al. 2017). In brief, 1 g of citric acid and 2 g of urea were dissolved in 20 mL of DMF to obtain a clear solution, and it was transferred into the 50 mL stainless steel for autoclave to be heated at 180 °C for 6 h hydrothermally. The brownish-red solution was obtained and diluted by 20 mL, 50 mg of NaOH solution. After the solution was centrifuged at 5000 rpm for 15 min, the dark solution was filtrated through 0.221 kDa dialysis membranes, through dialysis in double-distilled water for 48 h to remove unreacted compounds. Finally, it was lyophilized under –40 °C and stored for further functionalization and characterizations and the synthetic route of CQD preparation given in Supplementary Figure 1.

### 2.3. Synthesis of mono-6-deoxy-6-(*p-*tolyl sulfonyl)-β-cyclodextrin

Tosylate β-CD was prepared according to the previously reported method (Abdous et al. 2017). The 10 mmol β-cyclodextrin (11.34 g) was prepared in 100 mL of DD water and then the sodium hydroxide (1.2 g) in 3.6 mL of DD water was added in dropwise up to 6 min. Besides, the 10 mmol *p*-toluene sulfonyl chloride (1.943 g) was prepared in 15 mL of acetonitrile, and it was inserted into the β-CD solution mixture by slowly stirring for 2 h at room temperature 27 °C. Then, the reaction mixture was filtered and crystallized overnight at 4 °C. Further, the solution was neutralized with 10% HCl solution. Finally, the solution was washed with DD water and dried for ambient temperature for 24 h.

### 2.4. BOC protected glutamine

The procedure was followed by the earlier report with slight modifications (Heimer et al., 1983). Briefly, 0.0431 g of glutamine was taken and dissolved in 5% sodium hydroxide in a round bottom flask, following 0.0545 g of BOC anhydride was made in 20 mL of tetrahydrofuran and mixed with the glutamine solution. The mixture was then stirred for 6–7 h in a magnetic stirrer. Further, the solution was extracted with ethyl acetate three times. The organic layer was washed by water and brine solution was followed by the solution (organic layer) treated with sodium sulfate to remove any trace amount of water. The solution was then subjected to the rotatory evaporator and BOC protected Glu was removed for further reactions.

### 2.5. Synthesis of mono-(5-BOC-protected-glutamine-6-deoxy) β-cyclodextrin

Two hundred and fifty milligrams of tosylate β-CD was dissolved in 10 mL of DMF and stirred the solution by adding 100 mg of BOC protected glutamine at 75 °C for 4 h (Amarnath Praphakaran et al., 2018). The precipitate of the product was poured into 50 mL of ice-cold acetone and washed with acetone for five times to remove the unreacted BOC-protected glutamine. Obtained BOC glutamine-grafted-tosylate β-cyclodextrin was dried at 50 °C for three days in a hot air oven.

### 2.6. Conjugation of CQD with mono-(5-BOC-protected-glutamine-6-deoxy) β-cyclodextrin

Conjugation of CQD and mono-(5-BOC-protected-glutamine-6-deoxy) β-cyclodextrin (CQD-Glu-β-CD) was carried out through amidation reaction. Initially, 50 mg of CQD was dispersed in 10 mL of distilled water and then mixed with 1-ethyl-3-dimethyl aminopropyl carbodiimide (10 mg) and 10 mg of NHS to activate the carboxylic groups in the CQD, which forms a reactive ester by magnetic stirring at 2 h, further 50 mg of mono-(5-BOC-protected-glutamine-6-deoxy) β-cyclodextrin in 10 mL of water solution was added into the above mixture and magnetic stirring for 24 h at room temperature (27 °C). Finally, the obtained solution was dialyzed (MWCO 12,000) with DD water for 24 h, followed by lyophilization under –40 °C.

### 2.7. 5-Aminolevulinic acid-functionalized with CQD-Glu-β-CD

5-ALA was tagged with CQD functionalized mono-(5-BOC-protected-glutamine-6-deoxy) β-cyclodextrin via EDC/NHS chemistry. 5-ALA (100 mg) was activated by the reaction with 10 mg of 1-ethyl-3-(3-dimethyl aminopropyl) carbodiimide. Then, it was reacting with 10 mg of NHS to form a reactive ester group for 2 h reaction period. Further, 50 mg of CQD-Glu-β-CD was added to make a stable amide product between –COOH group of 5-ALA and –NH_2_ group of CQD-Glu-β-CD for 24 h reaction times at room temperature. Further, it was washed and dried by lyophilization under –40 °C.

### 2.8. Synthesis of DOX loaded 5-ALA-CQD-Glu-β-CD nanocarrier

**Figure 1.**
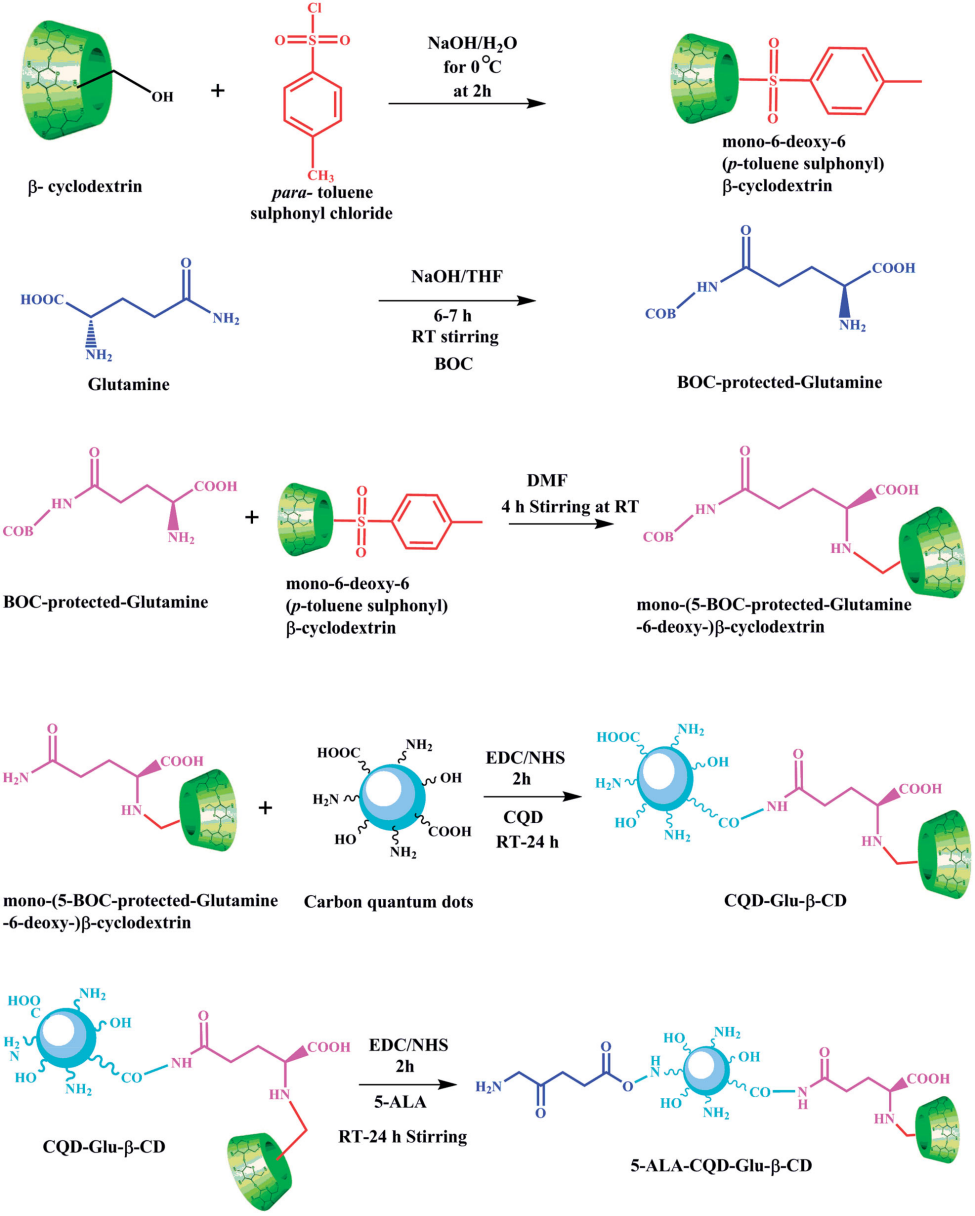
Overall representation for the preparation of 5-ALA-CQD-Glu-β-CD nanocarrier.

Fifty milligrams of 5-ALA tagged CQD conjugated BOC-glutamine-β-cyclodextrin was dissolved in 10 mL DD water, and 5 mg of DOX as anticancer drug was dissolved in 5 mL of ethanol, and then the drug solution was slowly added into the above carrier solution and magnetically stirred for 24 h under dark condition. Finally, an unreacted drug was removed through dialysis (MWCO 12,000) against DD water for 6 h at 27 °C. Further, the compound was freeze-dried through lyophilization at –40 °C. The overall schematic representation for the preparation of 5-ALA-CQD-Glu-β-CD nanocarrier presented in Figure 1.

### 2.9. Characterization studies

#### 2.9.1. Fourier transforms infrared spectroscopy analysis

Fourier transform infrared spectroscopy (Spectrum GX-1, Perkin Elmer, ‎Waltham, MA) was used to detect the chemical functional groups of (a) Ts-β-CD, (b) BOC-Glu, (c) Ts-β-CD-*g*-BOC-Glu, (d) CQD, (e) CQD-Glu-β-CD, (f) 5-ALA-CQD-Glu-β-CD, and (g) DOX/5-ALA-CQD-Glu-β-CD. It was prepared as pallet mixed with KBr and compressed to form a tablet. These tablets were scanned in the spectral region of 4000–400 cm^–1^.

#### 2.9.2. Transmission electron microscopy analysis

The morphology was characterized by transmission electron microscopy (TEM). For TEM study, a few droplets of as-prepared samples were placed on a carbon grid. The grids were examined under HRTEM, TECNAI F30, at a voltage of 80 kV.

#### 2.9.3. X-ray diffraction analysis

The crystalline nature of the prepared compounds was analyzed by using X-ray powder diffractometer (Philips 1710, Philips Electronic Instruments, Inc., Mahwah, NJ) with a copper target (Cu Kα1, *λ* = 1.54056 Å). It was worked a nickel filter at a voltage of 40 kV and a current of mA.

#### 2.9.4. Atomic force microscopy

Intermittent contact mode atomic force microscopy (AFM) (BT02218 Nanosurf, Liestal, Switzerland) was used to investigate the surface morphology of as-prepared compounds. The resonance frequency of the tip was retained between 146 and 236 kHz and force constant was kept at 48 N m^–1^.

#### 2.9.5. UV–visible, fluorescence, and Raman spectroscopy

The absorption intensity of as-synthesized carbon dots was analyzed by UV–vis spectroscopy (Shimadzu-1800, Kyoto, Japan). The emission range of carbon dots was measured using a spectrofluorometer (Shimadzu F-4500, Kyoto, Japan). The Raman spectra of carbon dots were investigated by Raman spectrometer (RAM HR 800) with a 632 nm excitation source of a He–Ne laser producing 17 mW power.

#### 2.9.6. Drug encapsulation efficiency and loading capacity analysis

Doxorubicin and 5-ALA were chosen as a model anticancer drugs and photo-modulator. DOX containing ethanol solution was gradually added to the 5-ALA-CQD-Glu-β-CD solution and 150 rpm for magnetic stirring at 27 °C. Then, the loading capacity was measured through (10 mg) of DOX/5-ALA-CQD-Glu-β-CD dispersed in 10 mL of DD water and ultrasonication for 15 min and centrifuged for 3000 rpm at 20 min. The supernatant solution was analyzed using UV–vis spectroscopy. Besides, EE and LC of 5-ALA and DOX were analyzed by UV–vis spectroscopy and absorption intensity of 5-ALA at the *λ*_max_ value of 253 and DOX *λ*_max_ value of 487 nm and calculated from the equation:

(1) $$\mathrm{EE}\;(\%)=\frac{\text{total amount of drug }–\text{ free amount of drug }}{\text{total amount of drug}}\times 100$$

(2) $$\mathrm{LC}\;(\%)=\frac{\text{total amount of drug }–\text{ free amount of drug }}{\text{weight of the dried nanocarrier}}\times 100$$

#### 2.9.7. *In vitro* drug release analysis

The drug release was evaluated using the dialysis method. In brief, as the required amount of DOX/5-ALA-CQD-Glu-β-CD nanocarrier was dissolved in 2 mL PBS solution in a dialysis tube, the solution containing a dialysis bag was immersed into 100 mL of PBS solution under magnetic stirring for 150 rpm at predetermined time intervals 2 mL throughout the PBS medium. Further, 2 mL of freshly prepared PBS medium was added. Finally, The drug-release percentage was detected using UV-Visspectroscopy (Shimadzu-1600) at the λ_max_ values 487 nm and 253 nm. 

### 2.10. Biological studies

#### 2.10.1. Cell culture

Human fibroblasts (WS-1) cell line and breast cancer (MCF-7) cell lines were purchased from National Centre for Cell Sciences (NCCS) (Pune, India). The cells were preserved in Dulbecco's modified Eagle medium (DMEM) supplemented with 10% fetal bovine serum and penicillin/streptomycin under 5% CO_2_ atmosphere at 37 °C.

#### 2.10.2. Determination of cell viability (MTT assay test)

The cytotoxic effect of fibroblast (WS-1) cell line, and breast cancer (MCF-7), cell lines were performed using tetrazolium salt of 3-[4,5-dimethylthiazol-2-yl]-2-5 diphenyltetrazolium bromide (MTT) assay. Both the cell lines were seeded with 96-well plates at a density of 1 × 10^5^ cells per well for 24 h at 37 °C. Different concentrations (0, 20, 40, 60, 80, and 100 μg/mL) of prepared compounds and free DOX were added into each well plate. After 24 h incubation, 0.5 μg/mL of MTT solution was added into each well; further, the cells were incubated with another 5 h at 37 °C. Afterward, a culture medium containing MTT was carefully removed and further added with 100 μg/mL of DMSO solution to dissolve a purple color formazan crystal. The optical density of each well plate was evaluated at 570 nm using an ELISA reader (enzyme-linked immunosorbent assay) microplate reader (BioTEK, Winooski, VT). Then, the IC_50_ concentration was measured compared to untreated cells.

IC_50_ calculation was followed by cell viability.

$$\text{Cell viability }(\%)=\frac{\text{OD of the treated cells}}{\text{OD of the untreated cells }(\mathrm{Control})}\times 100$$

OD mean value of the untreated cells (control).

#### 2.10.3. Photodynamic irradiation treatment

Continuous UV–visible light irradiation (SUV–VIS: 295–650 nm) was used for irradiation at a wavelength of 635 nm (25 mW cm^−2^) (Schott Glass, Mainz, Germany), after which cells were incubated for 3 min time intervals. Cells were irradiated in the dark from the top in an open culture dish containing 1 mL of fresh culture media.

#### 2.10.4. Detection of morphological changes analysis

Morphological changes and cell death of fibroblast (WS-1) cell line and breast cancer (MCF-7) cell line were performed using an inverted light confocal laser microscope (Olympus IX 81 under DU897 mode, Tokyo, Japan). Both cells were seeded with 96-well plates at a density of 1 × 10^5^ cells per well for 24 h. Then, the IC_50_ concentration level of DOX-loaded nanocarrier was added to both cell lines and incubated for 24 h. After 24 h incubation, morphological changes were observed using inverted light confocal laser microscopy at a wavelength of 570 nm.

#### 2.10.5. Hoechst 33342 staining analysis

Hoechst 33342 staining was used to study the cellular uptakes of the cells. The cells were seeded in 96-well plates at a density of 1 × 10^5^ cells/well for 24 h and then treated with different concentrations of DOX/CQD-Glu-β-CD, 5-ALA-CQD-Glu-β-CD, and DOX/5-ALA-CQD-Glu-β-CD nanocarrier. After further incubation for a particular time interval (6 h, 12 h, 18 h, and 24 h), the cells were washed with 1× PBS solution. Subsequently, the cells were stained with 1 μg/mL of Hoechst 33342 staining at 37 °C for 30 min under dark conditions. Then, the cell uptake was observed using a confocal laser microscope (Olympus IX 81 under DU897 mode, Tokyo, Japan).

#### 2.10.6. Qualitative cell uptake analysis

The MCF-7 cells were seeded with six-well plates at a density of 1 × 10^5^ cells per well for 24 h at 37 °C. One milliter of coumarin-6 loaded DOX/5-ALA-CQD-Glu-β-CD nanocarrier was added at an IC_50_ concentration of 60 mg/mL. After 12 h incubation period, the cells were washed with cold PBS and 4% paraformaldehyde and image was taken using blue filter (excitation wavelength 460–490 nm, emission 470 nm) in inverted fluorescence microscopy.

#### 2.10.7. Statistical analysis

The experiments were repeated at least three times expressed as a mean ± standard deviation using ANOVA. In all tests, statistical significance was set at *p < .05. 

## 3. Results and discussion

### 3.1. Fourier transform infrared spectroscopy analysis

**Figure 2.**
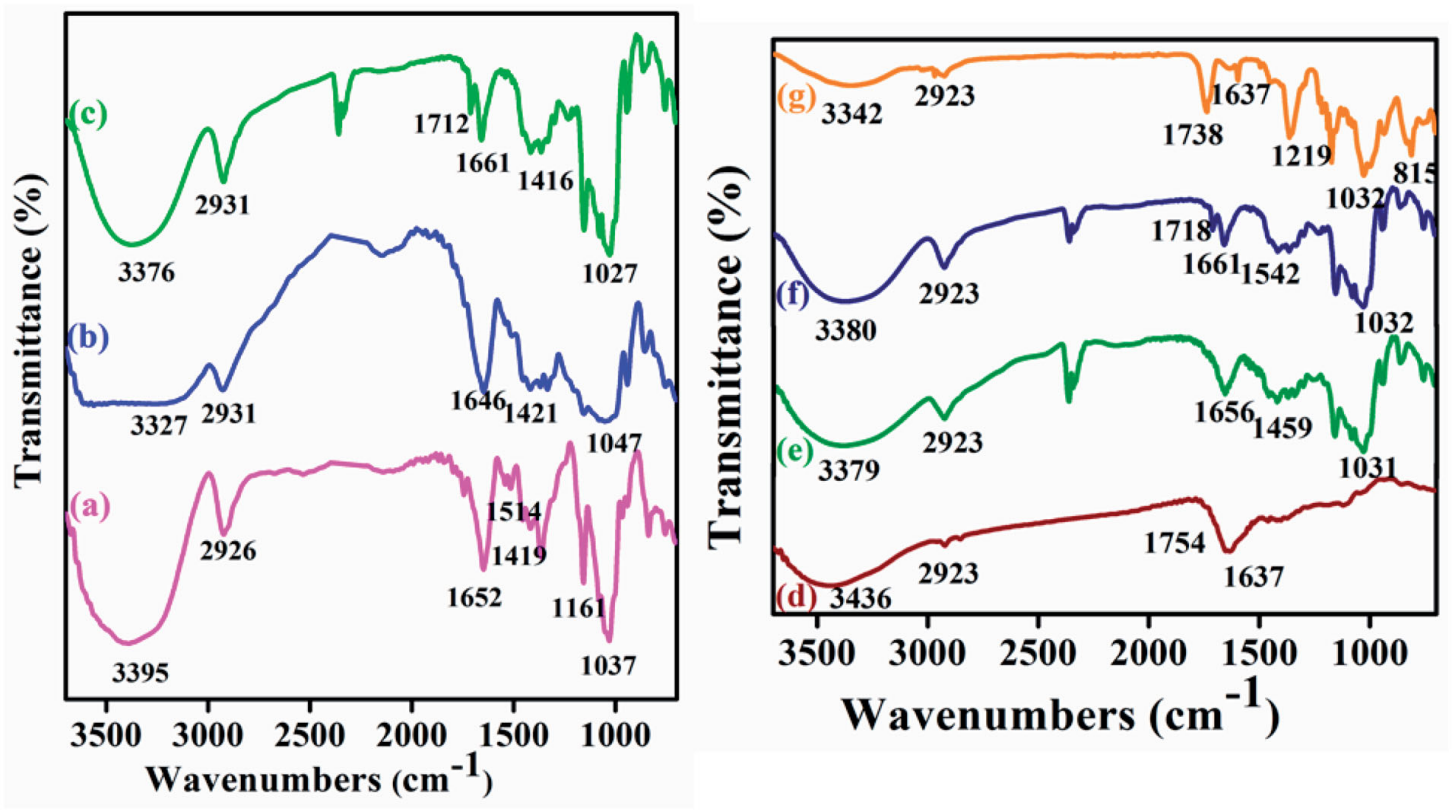
FTIR spectra of (a) Ts-β-CD, (b) BOC-Glu, (c) Ts-β-CD-*g*-BOC-Glu, (d) CQD, (e) CQD-Glu-β-CD, (f) 5-ALA-CQD-Glu-β-CD, and (g) DOX/5-ALA-CQD-Glu-β-CD nanocarrier.

The entire synthesized compounds were characterized through Fourier transform infrared (FT-IR) spectroscopy for the chemical functionality of the compounds. Figure 2 illustrates the FT-IR spectrum of (a) Ts-β-CD, (b) BOC-Glu, (c) Ts-β-CD-g-BOC-Glu, (d) CQD, (e) CQD-Glu-β-CD, (f) 5-ALA-CQD-Glu-β-CD, and (g) DOX/5-ALA-CQD-Glu-β-CD. In Figure 2(a), the sharp absorption peak at 3395 cm^–1^ corresponds due to the –OH stretching vibration of β-CD. The peak appeared at 1514 cm^–1^ represents the C=C stretching of the benzene ring in the β-CD structure. Besides, the frequency at 1419 cm^–1^ and 1161 cm^–1^ indicates the symmetric and asymmetric vibrations of the SO_2_ group of the tosyl group in β-CD (Pooresmaeil & Namazi, 2018). Figure 2(b) indicates the BOC protected glutamine which shows that the peaks at 1421 cm^–1^ and 1335 cm^–1^ are responsible for N–H stretching and C–O bending vibration of an amide bond by the successful protection of BOC in the glutamine moiety (Ju et al., 2011). Further, the BOC-Glu grafted with Ts-β-CD observed new peaks of carbonyl stretching (C=O) vibration at 1712 cm^–1^, then amino group (C–N) stretching vibration at 1661 cm^–1^ as given in Figure 2(c). The broad peak 3376 cm^–1^ indicates the N–H stretching vibration, which also confirms the existence of a glutamine molecule (Amarnath Praphakaran et al., 2018). The FT-IR spectrum of CQD presented in Figure 2(d) CQD exhibits a broad spectrum range at ∼3436 cm^–1^ due to O–H stretching vibration. The peaks that appeared at 2923 cm^–1^ indicate C=C stretching vibration, ∼1754 cm^–1^ corresponding to C=O, and 1637 cm^–1^ attributed to N–H bending vibration of CQD. The Ts-β-CD-*g*-BOC-Glu functionalized with CDQ exhibited the FTIR spectrum at 1656 cm^–1^ (–CO-NH–) which manifest the presence of amide-I bonds. The conjugation of the CQD and Ts-β-CD-g-BOC-Glu was an evidence of the new peak formations and shifting of lower frequency. After the conjugation of CQD and Ts-β-CD-g-BOC-Glu through amide bond formation, the secondary amine peak observed at 3379 cm^–1^ in Figure 2(e). The shifting of the peak from 3562 cm^–1^ to 3379 cm^–1^ is the new amide bond formation. Besides, the new peak attributed at 1031 cm^–1^ is due to the C–N stretching frequency for the conjugation of the CQD and Ts-β-CD-g-BOC-Glu through the amide bond (Xuewei et al., 2017). The successful functionalization of Ts-β-CD-g-BOC-Glu with CQD was noticed and presented in Figure 2(e). The photosensitizer of 5-ALA was conjugated with CQD-Glu-β-CD via EDC/NHS coupling reaction, which formed –CONH bond between –COOH groups of 5-ALA and –NH_2_ groups of CQDs. The peak at 1542 cm^–1^ corresponds to the N–H bending vibration of amide-II bonds given in Figure 2(f). Final DOX loaded CQD-based β-CD nanocarrier spectrum is illustrated in Figure 2(g). The characteristic peak of β-CD at 1652 cm^–1^ and 1037 cm^–1^ was shifted to 1738 cm^–1^ and 1027 cm^–1^ region, which indicates the presence of hydrogen bonding between the DOX and β-CD unit. Other peaks occurred at ∼815 cm^–1^ and ∼1219 cm^–1^ C–N stretching and N–H bending vibration of the DOX molecule in the nanocarrier system (Nasrollahi et al., 2016).

### 3.2. X-ray diffraction studies analysis

**Figure 3.**
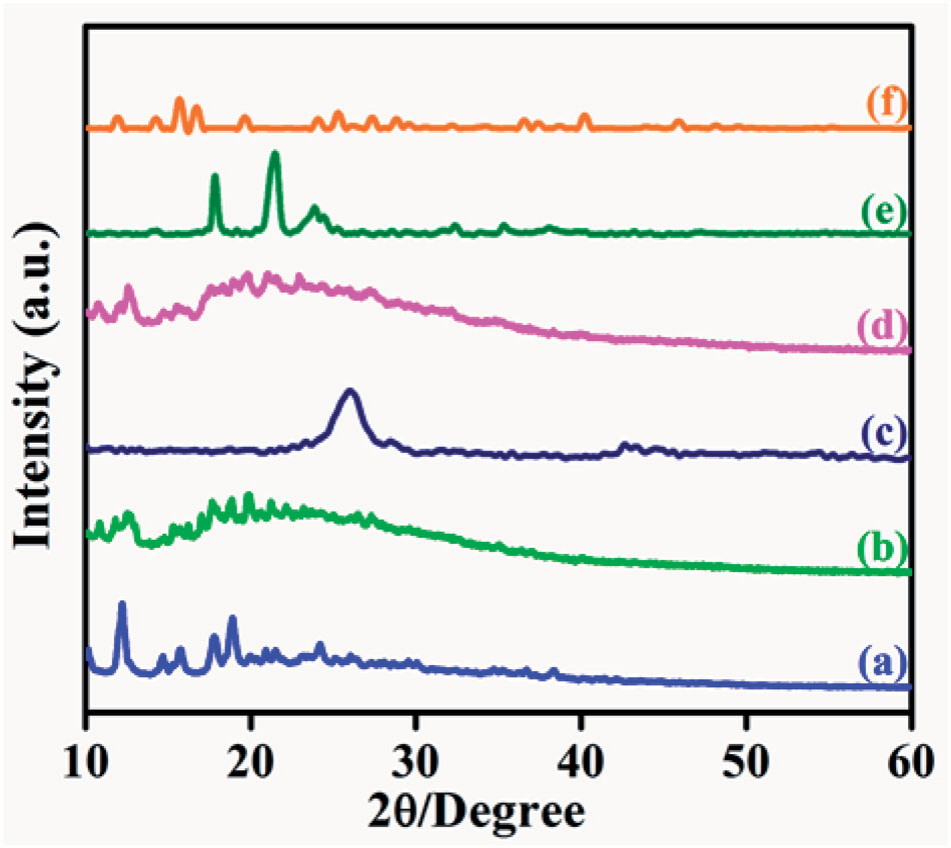
XRD pattern of (a) Ts-β-CD, (b) BOC-Glu, (c) Ts-β-CD-*g*-BOC-Glu, (d) CQD (e) CQD-Glu-β-CD, (f) 5-ALA-CQD-Glu-β-CD, and (f) DOX/5-ALA-CQD-Glu-β-CD nanocarrier.

The crystalline nature of as-synthesized compounds was analyzed by using X-ray diffraction (XRD) technique. The XRD pattern of Ts-β-CD gives several sharp peaks at 12.05 Å, 17.77 Å, and 18.84 Å which exhibits their crystalline behavior (Figure 3(a)). The XRD pattern of the BOC-Glu spectrum shows amorphous nature, and it was manifested in Figure 3(b). The XRD shape of CQD is represented in Figure 3(c), which observed a sharp peak at a 2*θ* value of 26.01 Å related to graphitic interlayer spacing in 3.2 Å. It reveals that more disorder graphitic carbon materials were formed (Liu et al., 2018). Figure 3(d) designates CQD-Glu-β-CD pattern of XRD to have multiple amorphous peaks and intensity of first CQD peaks has decreased, which indicates the successful conjugation of CQDs in BOC-Glu-*g*-β-CD. After tagging of 5-ALA in CQD-Glu-β-CD, 5-ALA corresponds due to two new sharp peaks appearing at 17.89 Å and 21.31 Å identified. CQDs peaks are red-shifted on 26.01–23.88 Å in Figure 3(e), which indicates the successful conjugation of 5-ALA with CQD moiety. The DOX was loaded with 5-ALA-CQD-Glu-β-CD nanocarrier which shows some prominent peaks at 15.70, 16.56, 24.04, and 27.46 Å obtained, which reveals semi-crystalline nature was due to loading of DOX through hydrogen bonding interaction and given in Figure 3(f) (Onodera et al., 2013).

### 3.3. Transmission electron microscopy analysis

**Figure 4.**
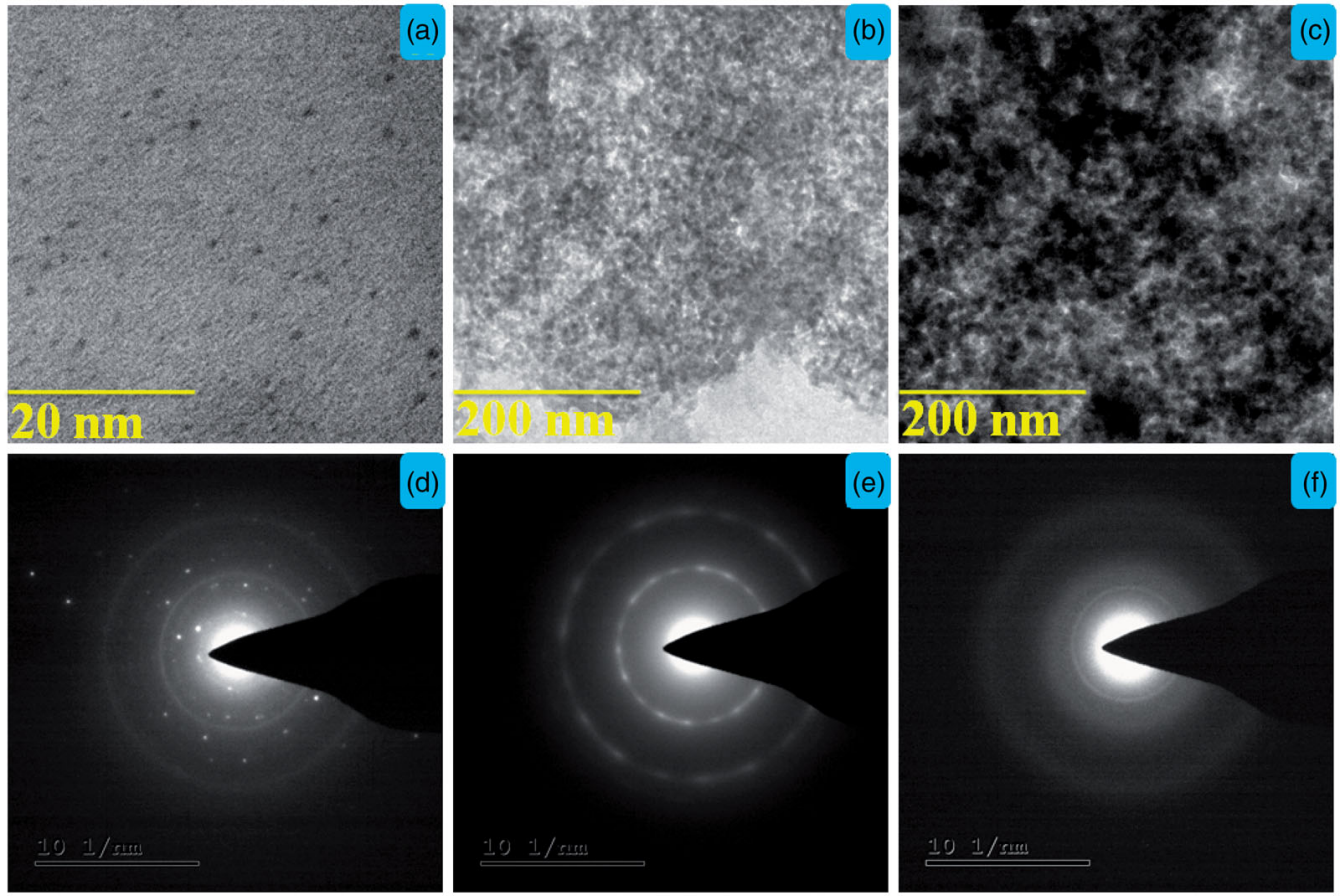
TEM images with SEAD spectrum of (a) CQD, (b) 5-ALA-CQD-BOC-Glu-β-CD, and (c) DOX/5-ALA-CQD-BOC-Glu-β-CD nanocarrier.

**Figure 5.**
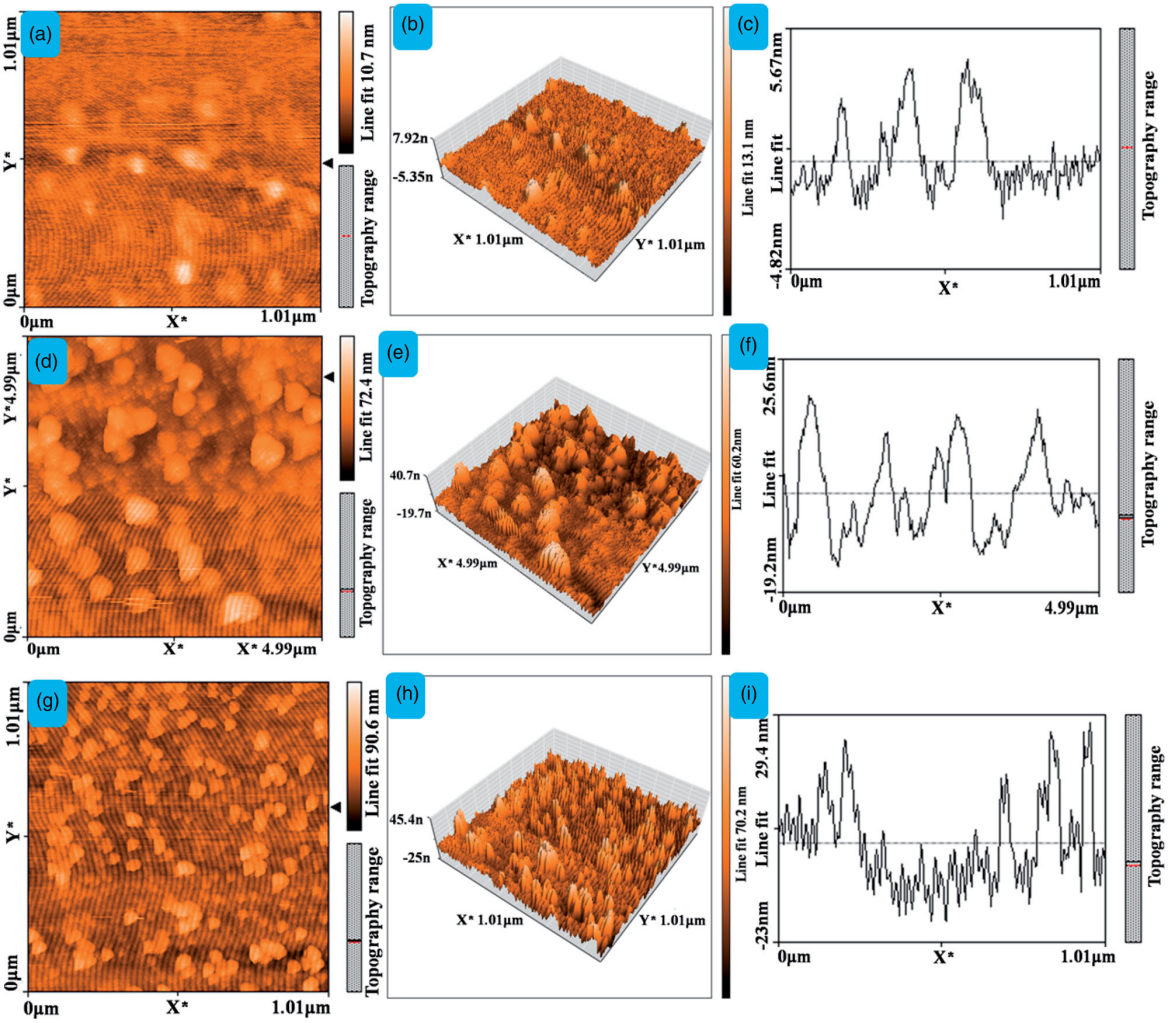
The AFM images of 2D, 3D, and topographical images of (a–c) CQD, (d–f) 5-ALA-CQD-BOC-Glu-β-CD, and (g–i) DOX/5-ALA-CQD-BOC-Glu-β-CD nanocarrier.

The morphological structures of (a) CQD, (b) 5-ALA-CQD-Glu-β-CD, and (c) DOX/5-ALA-CQD-Glu-β-CD were investigated using TEM analysis, and it is presented in Figure 4(a–c). The TEM images of CQD were shown as a well-known uniform spherical like structure (Figure 4(a)) and the SAED pattern of CQD reveals white spots of the ring which indicates good crystalline nature of the in-plane carbon materials (Figure 4(d)) with correlation of CQD-XRD pattern (Suzuki et al., 2017). After the surface modification of 5-ALA-CQD-Glu-β-CD, the size was increased ∼200 nm (Figure 5(b)) and the SAED pattern indicates some white dots corresponding to the semi-crystalline polysaccharides core (Figure 4(e)). Finally, the DOX loaded 5-ALA-CQD-Glu-β-CD via host–guest and hydrophobic interaction between the two molecules was observed with a profoundly dark intense particle with spherical morphology and SAED pattern of DOX-loaded nanocarrier shows amorphous nature and given in Figure 4(c,f).

### 3.4. Atomic force microscopy analysis

AFM studies observed the surface topography of as-synthesized (a) CQD, (b) 5-ALA-CQD-BOC-Glu-β-CD, and (c) DOX/5-ALA-CQD-BOC-Glu-β-CD nanocarrier. AFM images were corresponding to surface range, and height profile was exposed in Figure 5. Figure 5(a–c) depicts the CQDs are spherical in structure, and the average size was observed at ∼5.67 nm. Our synthesized carbon dots size was correlated with previous report of CQDs by the preparation using citric acid and urea as the precursors to obtain the same spherical like structure (Ding et al., 2017). The AFM morphological observation of 5-ALA tagged CQD grafted BOC-protected Glu-β-CD is given in Figure 5(d–f), and cold lumps like shape particles were appeared on the surface. The surface roughness also increased the size ∼25.6 nm. The DOX molecules loaded on the hydrophobic cavity of β-CD are displayed in Figure 5(g–i) and results show the increases of the roughness to a size range ∼29.4 nm. The results have a good connection with the report of polymer functionalized graphene quantum dot-based system, and the thickness was increased after the encapsulation of DOX in the system (De et al., 2018).

### 3.5. UV–visible, fluorescence, and Raman spectroscopy

UV–vis spectra of citric acid derived fluorescent CQD observed two absorption bands at 252 and 313 nm. It is presented in Supplementary Figure 2(a). The first absorption band was attributed at π–π* electronic transition of C=C bonds of sp^2^ carbon domains and next absorption band is n–π* electronic transition of C=O group of carbon dot (Zhou et al., 2015). Supplementary Figure 2(b) demonstrates CQD has strong green fluorescence in the water at the emission range of 485 nm in UV–vis spectroscopy and excitation wavelength of carbon dot at *λ*_max_ value at 380 nm. Inset images of Supplementary Figure 2(b) are the CQDs solution in light and emitting green color fluorescence under UV-light irradiation (D’Souza et al., 2018). The Raman spectrum of CQD was presented in Supplementary Figure 2(c) and two influential bands as D at 1346 cm^–1^ and G at 1580 cm^–1^ were noted. The D band reveals that A_1g_ mode of disorder plane of carbon materials and G band corresponds due to the E_2g_ way of carbon mode of sp^2^ hybridized carbon domains, respectively (Chen et al., 2018).

### 3.6. Determination of drug encapsulation and loading properties

**Figure 6.**
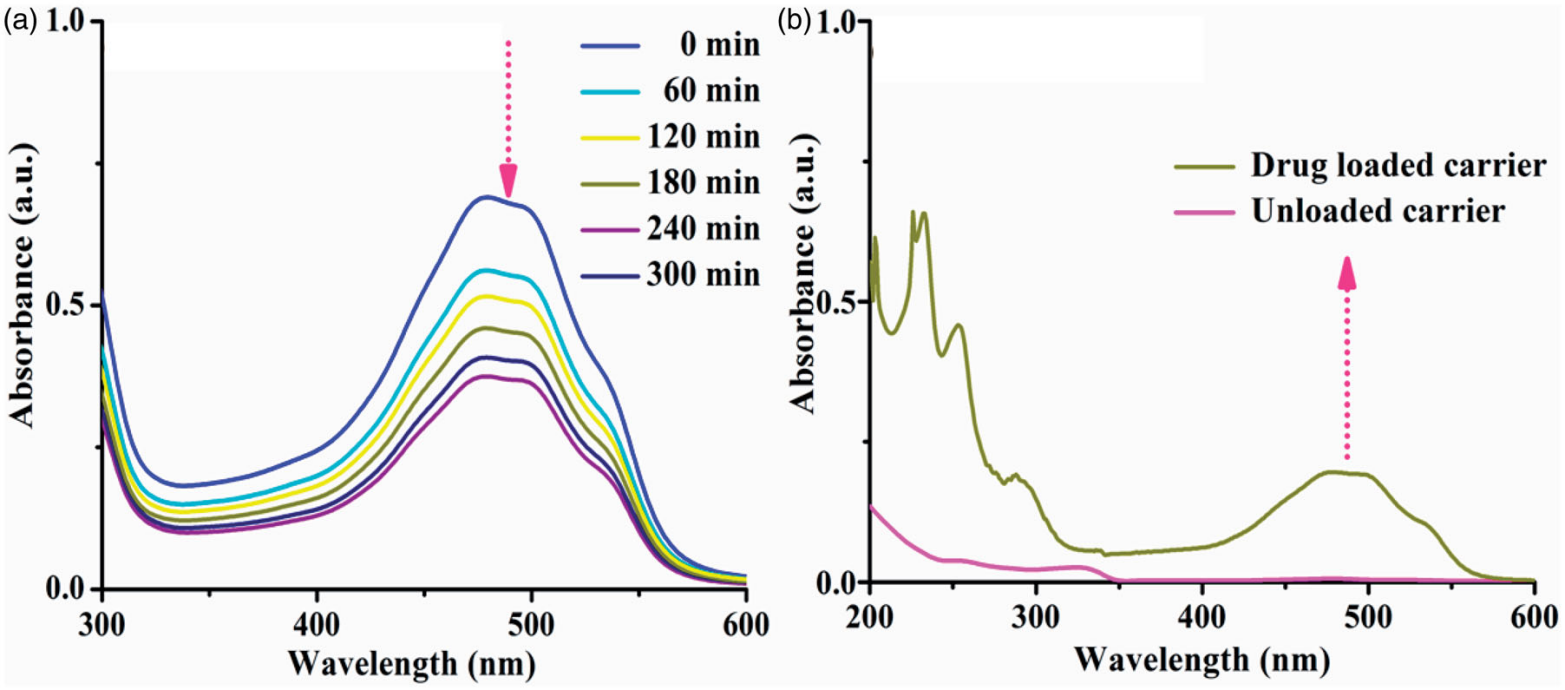
UV–vis spectrum (a) encapsulation efficiency and (b) loading capacity of DOX/5-ALA-CQD-BOC-Glu-β-CD nanocarrier.

The drug encapsulation efficiency and loading capacity are the most crucial role of the drug delivery system under examination drug carrier system in different physiological conditions, and DOX loaded system was investigated (Figure 6(a,b)). The DOX was effectively encapsulated on the lipophilic cavity of β-CD, through host–guest interactions, hydrophobic, and hydrogen-bonding interactions after mixing with the 5-ALA-CQD-BOC-Glu-β-CD carrier (Shen & Qiu, 2018). The absorption intensity of DOX was decreased with increasing time intervals, which indicates drug molecules effectively encapsulated on the hydrophobic cavity of β-CD. The encapsulation efficiency of the DOX drug is ∼83.0% and the result of the spectrum is given in Figure 6(a). DOX/5-ALA-CQD-Glu-β-CD loading capacity was investigated by the vortex for 30 min and the loading capacity of 5-ALA and DOX was observed at ∼18.15% and ∼20.37% correspondingly and given in Figure 6(b).

### 3.7. *In vitro* drug-releasing studies

**Figure 7.**
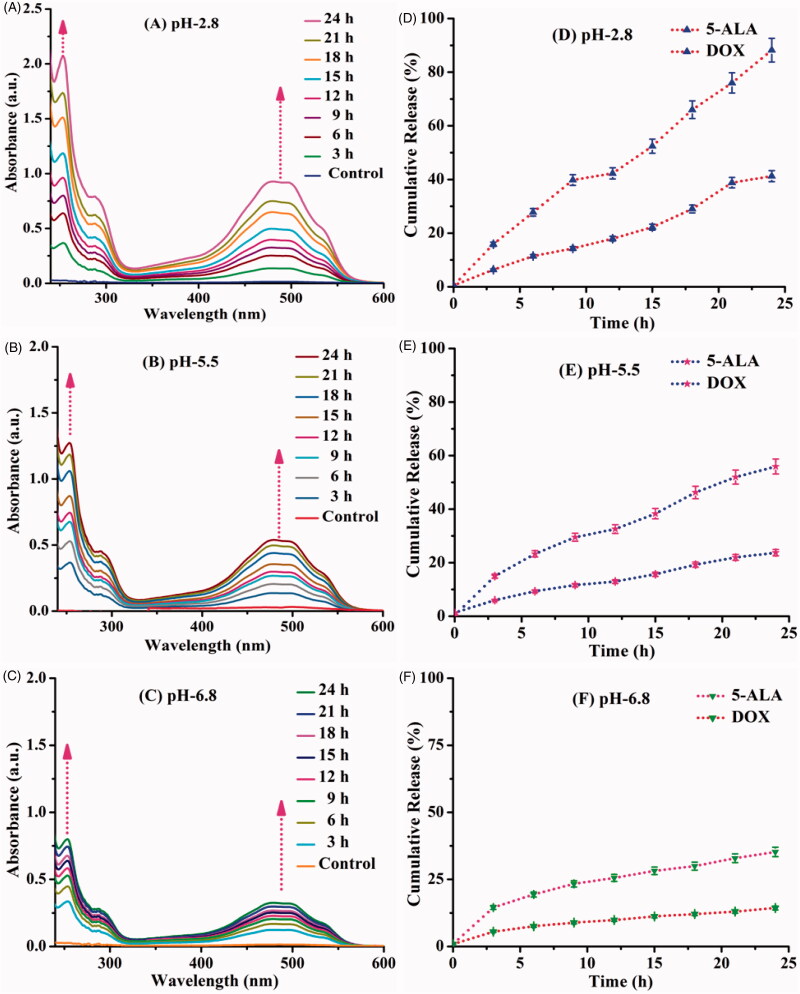
*In vitro* drug release behavior of 5-ALA and DOX drug from DOX/5-ALA-CQD-BOC-Glu-β-CD under three different physiological pHs (A) 2.8, (B) 5.5, (C) 6.8 at 27 °C for 24 h (A–C). Cumulative drug release (%) behavior (D–F).

The 5-ALA and DOX are releasing behaviors determined in a different physiological condition such as pH 2.8, pH 5.5, and pH 6.8 at room temperature (27 °C), and the drug release pattern is given in Figure 7. The drug release was mainly influenced by the swelling of the carrier based on the pH of the solution, the amide linkage of CQD, and 5-ALA easily broken in acidic medium and the amine groups of DOX quickly protonated in acidic pH. Since the carrier was easily swelled in acidic pH and released high rate of 5-ALA of ∼91%, DOX is ∼41.25% in pH 2.8. In the pH 5.5 and 6.8 medium, the swelling behavior of the carrier was less due to the cleavage of the amide bond and deprotonating of amine groups was somewhat difficult at this condition. Due to less amount, the drug release rate was observed in pH 5.5 and 6.8 compared with pH 2.8. The release rate of pH 5.5 and pH 6.8 exhibits 5-ALA was ∼55.94%, ∼32.90% and DOX ∼23.27%, ∼13.0%, respectively. The pH influenced the release of the drug since the carrier behavior has significant advantages through cancer cell microenvironment in acidic nature surroundings in cancer cells (Vinothini et al., 2019).

### 3.8. *In vitro* cell cytotoxicity studies

**Figure 8.**
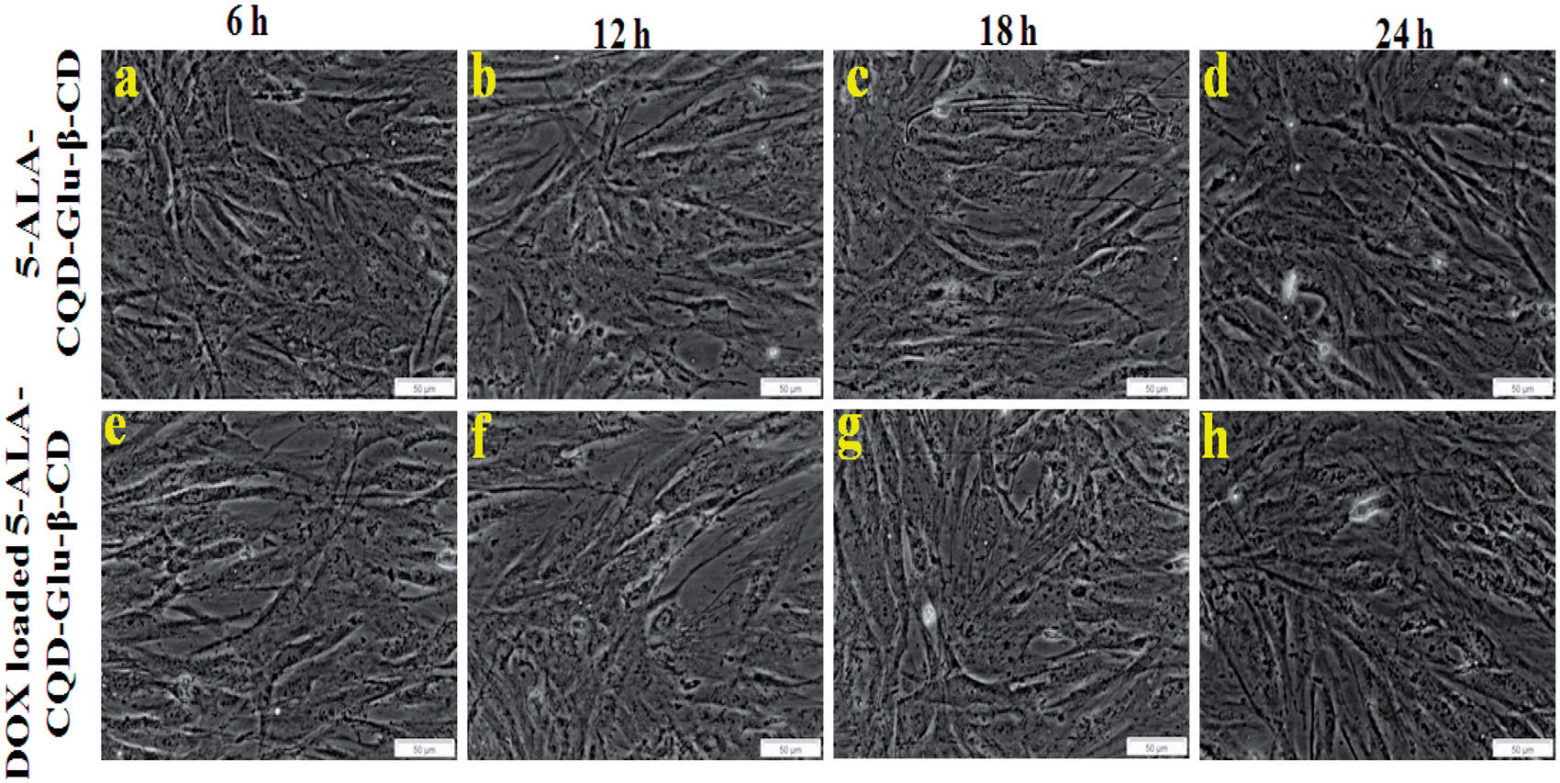
*In vitro* morphological changes of fibroblast (WS-1) cell line treated with 5-ALA CQD-Glu-β-CD and DOX/5-ALA-CQD-Glu-β-CD nanocarrier in a various time-dependent manner such as 6 h, 12 h, 18 h, and 24 h and IC_50_ concentration of 60 μg/mL at scale bar: ∼50 μm.

**Figure 9.**
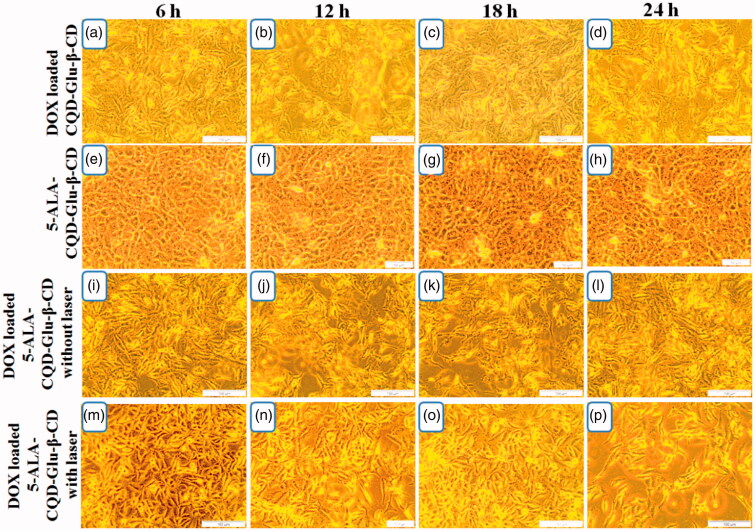
*In vitro* morphological changes by the chemo-photodynamic effect of breast cancer (MCF-7) cell treated with DOX/CQD-Glu-β-CD, 5-ALA-CQD-Glu-β-CD, and DOX/5-ALA-CQD-Glu-β-CD nanocarrier with and without irradiation in various time-dependent manners such as 6 h, 12 h, 18 h, and 24 h and IC_50_ concentration of 60 μg/mL at scale bar: ∼100 μm.

The cytotoxic effect of prepared compounds was evaluated in the fibroblast cell line (WS-1) and breast cancer cell line (MCF-7) by MTT assay method. The quantitative amount of cell viability of WS1 and MCF-7 is given in Supplementary Figure 3(a,b). Supplementary Figure 3 presents the cell viability of free DOX, free 5-ALA, CQD-Glu-β-CD, 5-ALA-CQD-Glu-β-CD, and DOX/5-ALA-CQD-Glu-β-CD (with and without irradiation) on fibroblast (WS-1) cell line with different concentrations (0, 20, 40, 60, 80, and 100 μg/mL) at 24 h time intervals. In this analysis, free DOX shows lower cell viability effect, and other samples 5-ALA, CQD-Glu-β-CD, and 5-ALA-CQD-Glu-β-CD exhibit no noticeable cytotoxic effect, and it shows good biocompatibility nature against the WS1 cells. Similarly, the DOX/5-ALA-CQD-Glu-β-CD treated MCF-7 cell shows a higher amount of cytotoxicity at a 24 h period with and without irradiation than free DOX drug. The cytotoxic effect increased with increasing concentration of DOX/5-ALA-CQD-Glu-β-CD nanocarrier, and the IC_50_ concentration level of 45.1% was observed at 60 μg/mL in MCF-7 cells, and the results are given in Supplementary Figure 3(b). Sixty micrograms per milliliter concentration of DOX/5-ALA-CQD-Glu-β-CD nanocarrier further used for different time interval investigation and cell morphology was represented in Figures 8 and 9 for WS-1 and MCF-7 cells, respectively. No significant morphological changes of WS1 were observed with increasing incubation time with 5-ALA-CQD-Glu-β-CD and DOX/5-ALA-CQD-Glu-β-CD. These results indicate the 5-ALA-CQD-Glu-β-CD and DOX/5-ALA-CQD-Glu-β-CD carrier have good viable nature against WS-1 cells. Likewise, the DOX/CQD-Glu-β-CD, 5-ALA-CQD-Glu-β-CD, and DOX/5-ALA-CQD-Glu-β-CD nanocarrier (with and without irradiation) treated on MCF-7 cells and morphological changes were observed by an optical microscope as given in Figure 9. All the results illustrate that the cell damages were more severe at a more extended period of incubation at 24 h. From Figure 9(i–l), the cancer cells were mainly affected by DOX/5-ALA-CQD-Glu-β-CD for 24 h of incubation period when compared with 5-ALA-CQD-Glu-β-CD and DOX/CQD-Glu-β-CD nanocarrier. The synergistic effect of DOX and 5-ALA alone shows only smaller amount of cell damages observed, as presented in Figure 9(a–d, e–h). More considerable cell damage was obtained on increases by irradiation of DOX/5-ALA-CQD-Glu-β-CD incubated cell at a 24 h period. The higher cell damaged morphology was observed in the DOX/5-ALA-CQD-Glu-β-CD nanocarrier with laser irradiation, and it is represented in Figure 9(m–p). 5-ALA generates more amount of ROS during the laser irradiation period, and inhibits the cancer cell with a combination of DOX drug by the synergistic effects of chemo and photodynamic impact (Gabas et al., 2016).

### 3.9. *In vitro* live and ROS generation analysis

**Figure 10.**
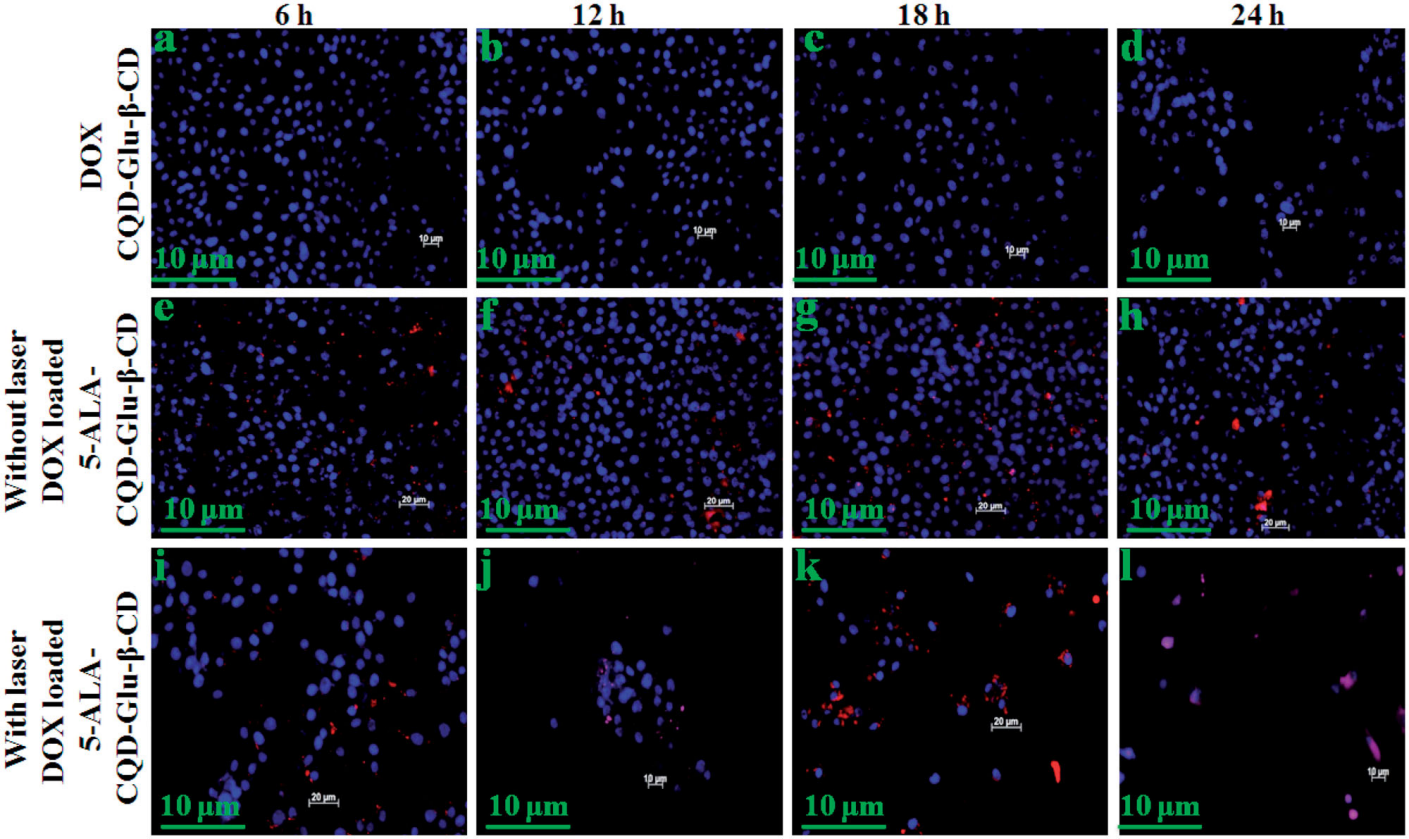
Fluorescent microscopic images of breast cancer (MCF-7) cell line incubated with DOX-loaded CQD-Glu-β-CD, and DOX-loaded 5-ALA-CQD-Glu-β-CD nanocarrier without and with laser irradiation in a various time-dependent manner such as 6 h, 12 h, 18 h, and 24 h and IC_50_ concentration of 60 μg/mL respectively at scale bar: ∼30 μm.

**Figure 11.**
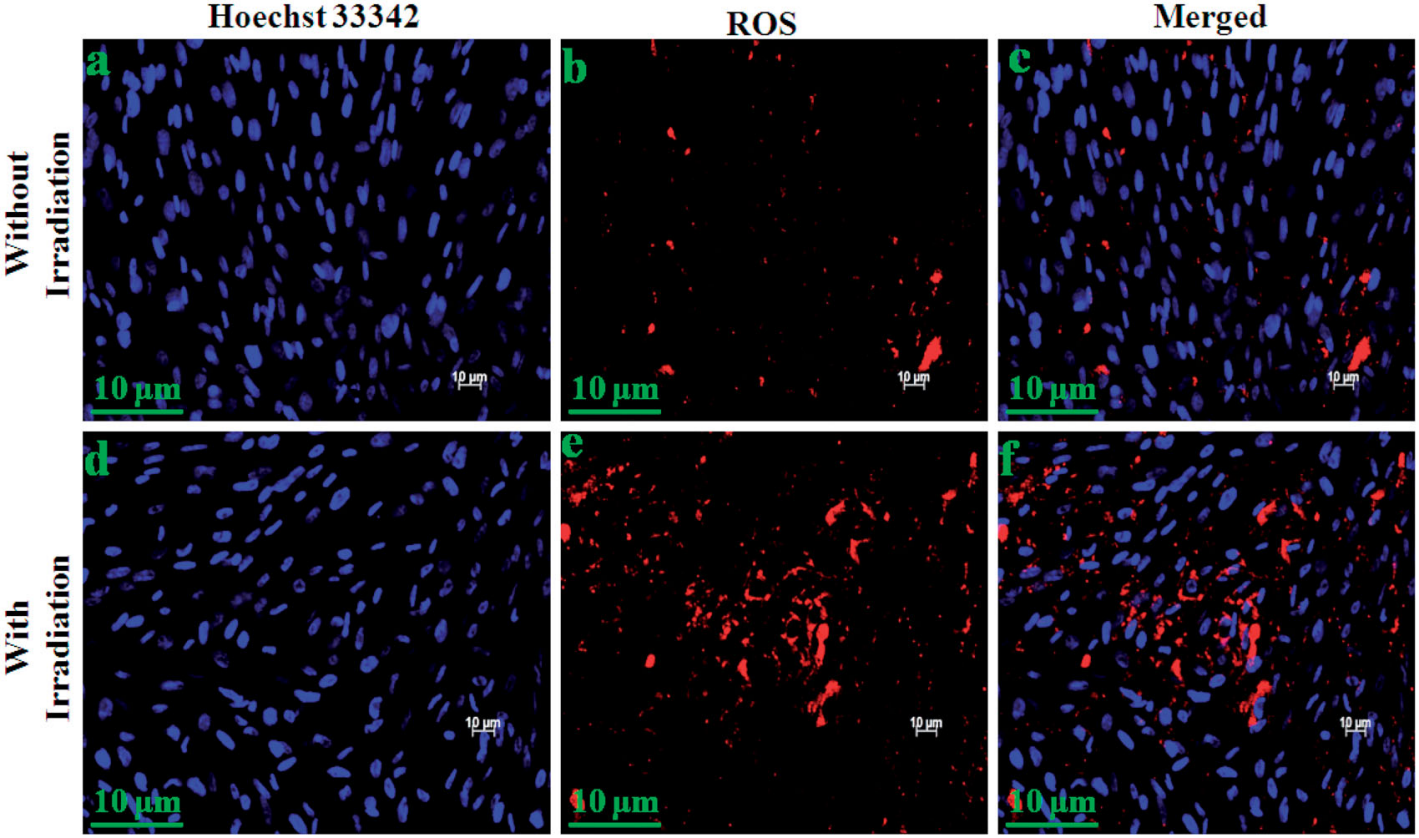
ROS level evaluation under irradiation in 6 h incubation with DOX/5-ALA-CQD-Glu-β-CD nanocarrier in breast cancer (MCF-7) cell line without (a–c) and with radiation (d–f) scale bar: ∼10 μm.

The live and ROS generation of DOX/CQD-Glu-β-CD, and DOX/5-ALA-CQD-Glu-β-CD nanocarrier (without and with laser irradiation) was investigated in MCF-7 cell line via fluorescent microscopy. The cancer cell exhibits an increased cell uptake of the anticancer drug leading to cell death which was observed with treatment of DOX/CQD-Glu-β-CD and DOX/5-ALA-CQD-Glu-β-CD nanocarrier by various time-dependent manners. Cell death and cell shrinking were found with the increasing time hours with the IC_50_ concentration. Laser combined DOX/5-ALA-CQD-Glu-β-CD demonstrated that a higher amount of cell death is due to the synergetic effects of mixed anti-therapeutics drug and photo-sensitizer. The laser without the DOX/5-ALA-CQD-Glu-β-CD system exhibits lower toxicity effects against the MCF-7 cancer cells. Numbers of evidence reported the synergistic impact of chemo-photosensitizer for the inhibition of cancer cells (Hu et al., 2015). Synergistic effect in cancer cell inhibition was observed by the intercalation of DNA and disruption of topoisomerase-II-mediated DNA repair and ALA-mediated PpIX, besides in cancer cells and sufficient cell death observed after irradiation (Babic et al., 2018). In comparison with those treated with DOX/CQD-Glu-β-CD nanocarrier, the cells were incubated with DOX/5-ALA-CQD-Glu-β-CD without laser which they did not produce ROS as given in Figure 10(e–h). Besides, laser-irradiated DOX/5-ALA-CQD-Glu-β-CD nanocarrier treated with breast cancer MCF-7 cell line has a maximum nucleus cell death compared to DOX/CQD-Glu-β-CD as presented in Figure 10(i–l). Further, the basic concept of PDT is the generation of highly cytotoxic singlet oxygen under laser irradiation. Also, Figure 11(a–c) demonstrates that DOX/5-ALA-CQD-Glu-β-CD nanocarrier does not produce any ROS with irradiation and lower toxicity in MCF-7 cells. Then, the laser combined DOX/5-ALA-CQD-Glu-β-CD nanocarrier shows more amount of ROS generation as it is represented in Figure 11(d–f). The combined DOX/5-ALA-CQD-Glu-β-CD drug carrier rapidly and massively was endocytosed into the cells under laser condition. In contrast, the intercalation of DNA and PpIX molecules produced more amount of cell death by disruption of topoisomerase-II-mediated DNA and repair ROS level on the cancer cell line.

### 3.10. *In vitro* cell uptake analysis

**Figure 12.**
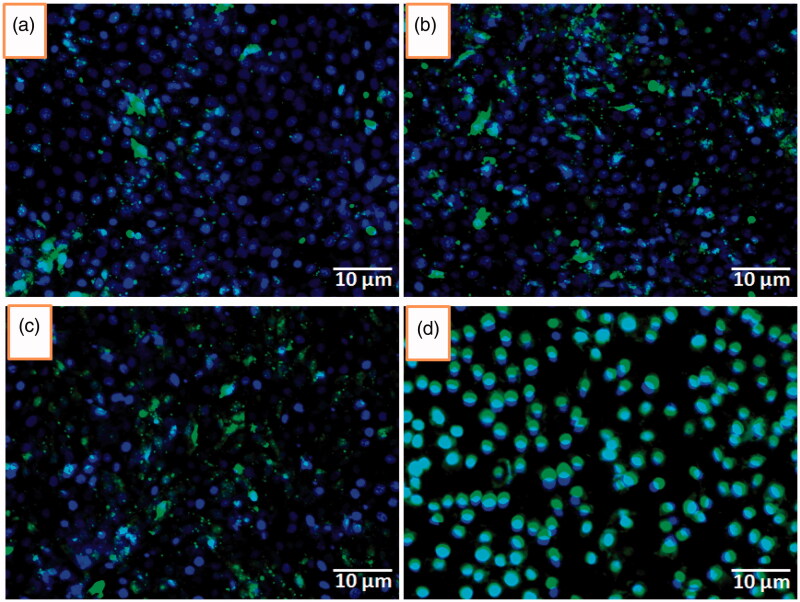
Qualitative assessment of fluorescence intensity associated with MCF-7 cells by the incubation of coumarin-6 loaded CQD-Glu-β-CD nanocarrier for 1 (a), 3 (b), 6 (c), and 12 h using blue filter (excitation wavelength 460–490 nm, emission 470 nm) in inverted fluorescence microscopy.

To evaluate the *in vitro* qualitative and quantitative cell uptake, the coumarin-6 tagged DOX/5-ALA-CQD-Glu-β-CD was studied in a different time-dependent manner using fluorescence microscopy as given in Figure 12. The investigation demonstrates the localization of the DOX/5-ALA-CQD-Glu-β-CD nanocarrier MCF-7 cell. The fluorescence intensity increased with an increase in the incubation time, most probably due to the increased uptake and the subsequent release of DOX from the nanocarrier. The intracellular uptake with increasing of time hours, at 12 h maximum drug-loaded carrier, was uptaken by the MCF-7 cells.

## 4. Conclusions

Fluorescent-based CQD with photosensitizer functionalized and chemo drug-loaded nanocarrier system was prepared. The prepared materials such as CQD, Ts-β-CD, BOC-Glu, CQD-Glu-β-CD, 5-ALA-CQD-Glu-β-CD, and DOX/5-ALA-CQD-Glu-β-CD nanocarrier were characterized by FTIR, XRD, TEM, and AFM, etc. The excellent surface morphological observation observed the as-synthesized materials described through TEM and AFM techniques. The encapsulation efficiency of DOX is ∼83.0% which found using UV–vis spectroscopy. An *in vitro* drug release study shows that the release pattern of DOX from DOX/5-ALA-CQD-Glu-β-CD denoted the carrier as pH-dependent drug release in profile and more releases were observed at acidic pH 2.8, ∼91% of 5-ALA and ∼41.25% at 24 h time intervals. The photodynamic effect of ROS generation leads to cell damage and morphological changes of cells analyzed against breast cancer MCF-7 cell line. The laser-treated DOX/5-ALA-CQD-Glu-β-CD nanocarrier manifested superior physico-chemical properties, controllable, and pH-dependent drug-releasing behaviors were successfully reported. Thus, the synergetic roles of DOX/5-ALA-CQD-Glu-β-CD demonstrate with the high potential and combined nano-drug delivery for cancer treatment.

## Supplementary Material

Supplemental Material
