# Supplementary material for: Combined photodynamic-chemotherapy investigation of cancer cells using carbon quantum dot-based drug carrier system
Source: Drug Deliv. 2020 May 18;27(1):791–804. doi: 10.1080/10717544.2020.1765431 (PMC7301704; doi:10.1080/10717544.2020.1765431)
Supplement: Supplemental Material [file IDRD_A_1765431_SM1120.docx]

**Supplementary information**

**Combined Photodynamic-Chemotherapy investigation of Cancer Cells using Carbon Quantum Dot-Based Drug Carrier System**

**Xin Li^a^, Kandasamy Vinothini^bc^, Mariappan Rajan^c^*, Andy Ramu^b^***

^a^Department of Medical Oncology, Xinxiang Central Hospital,The Fourth Clinical College of Xinxiang Medical College, Xinxiang City,Henan Province, 453000, China

^b^Department of Inorganic chemistry, School of Chemistry, Madurai Kamaraj University, Madurai 625021, Tamil Nadu, India.

^c*^Biomaterials in Medicinal Chemistry Laboratory, Department of Natural Products Chemistry, School of Chemistry, Madurai Kamaraj University, Madurai 625021,Tamil Nadu, India.

***Corresponding author**

Mariappan Rajan, Biomaterials in Medicinal Chemistry Laboratory, Department of Natural Products Chemistry, School of Chemistry, Madurai Kamaraj University, Madurai - 625021, India. Tel: +91 9488014084; Fax: 0452-2459845; Email: [rajanm153@gmail.com](mailto:rajanm153@gmail.com).

**1. Carbon quantum dots methodology**

***
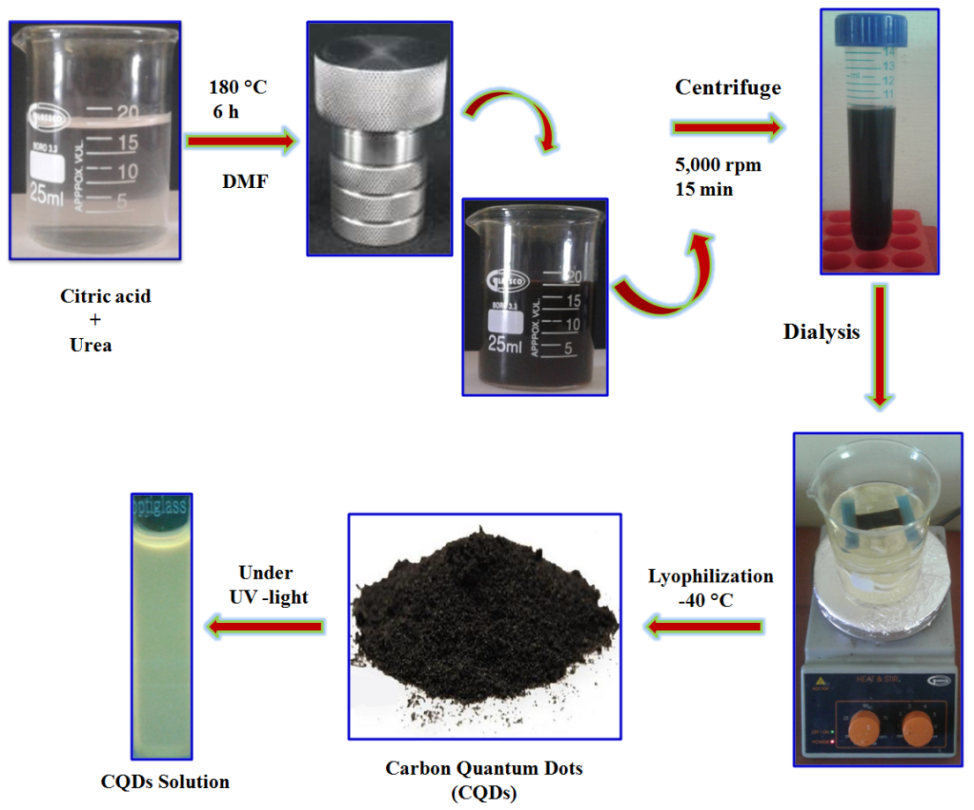
***

**S.Figure 1.** Synthetic representation of carbon quantum dot (CQD) preparation

**2. UV-Visible, Fluorescence and Raman spectroscopy**

***
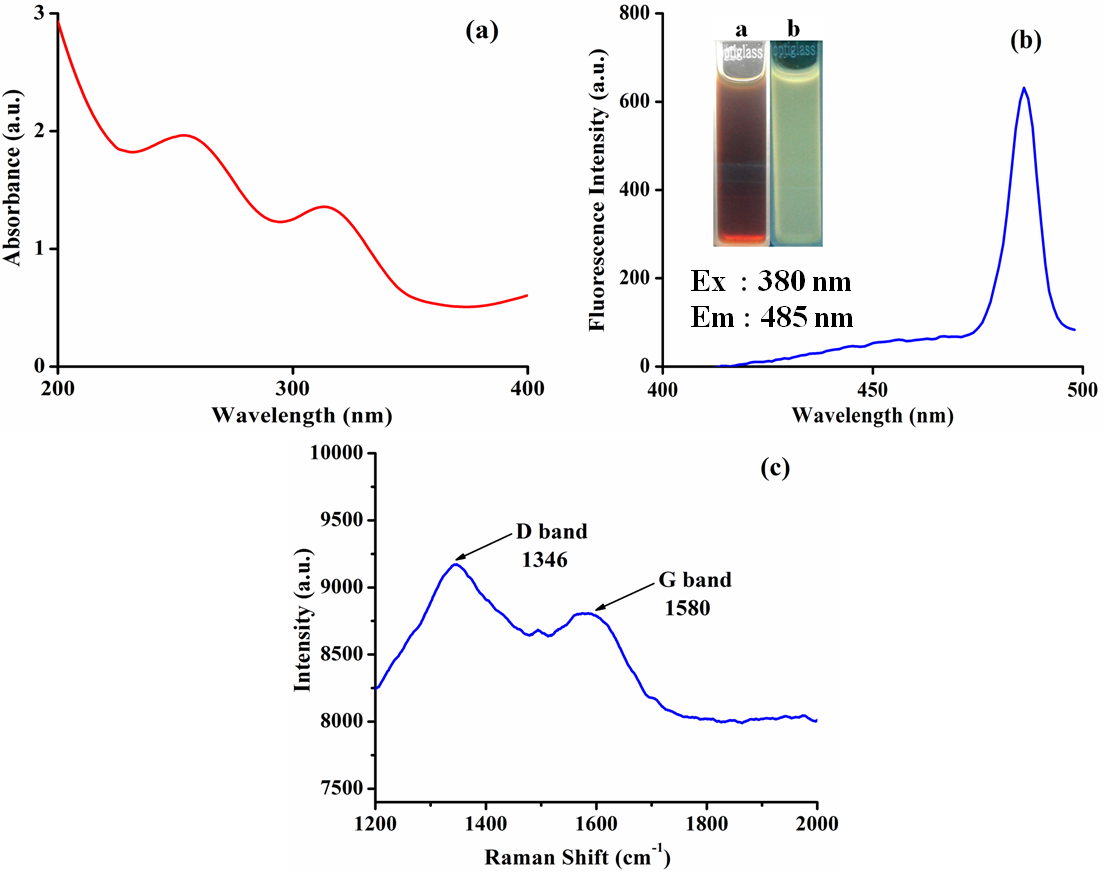
***

**S.Figure 2.** Carbon quantum dot spectrum of (a) UV-Visible spectroscopy (b) Fluorescence spectrum and (c) Raman Spectrum

**3. *In-vitro* cell cytotoxicity**


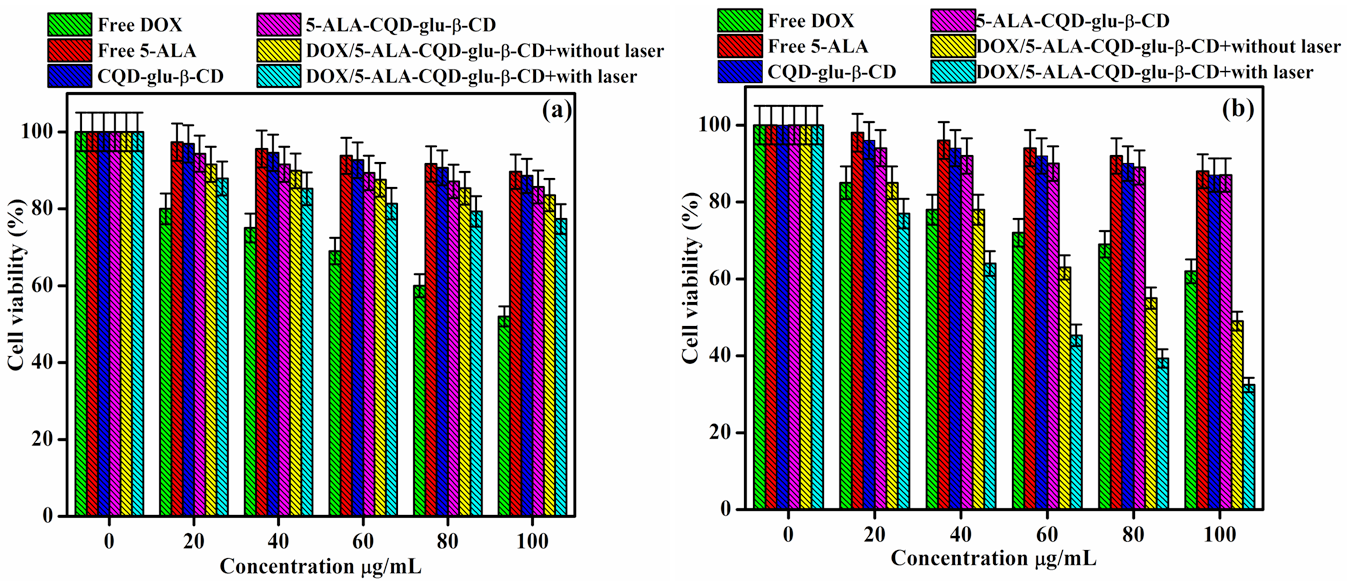


**S.Figure 3 (a&b).** *In-vitro* cytotoxic effect of fibroblast (WS-1) cell line and breast cancer (MCF-7) cell line in 24 h incubation with Free DOX, Free 5-ALA, CQD-Glu-β-CD, 5-ALA-CQD-Glu-β-CD and DOX/5-ALA-CQD-Glu-β-CD in without and with laser treatment at different concentration manner such as 0, 20, 40, 60, 80 and 100 μg/mL.
